# Organizational Health Literacy: Quality Improvement Measures with Expert Consensus

**DOI:** 10.3928/24748307-20190503-01

**Published:** 2019-07-01

**Authors:** Angela G. Brega, Mika K. Hamer, Karen Albright, Cindy Brach, Debra Saliba, Dana Abbey, R. Mark Gritz

## Abstract

**Background:**

Organizational health literacy (OHL) is the degree to which health care organizations implement strategies to make it easier for patients to understand health information, navigate the health care system, engage in the health care process, and manage their health. Although resources exist to guide OHL-related quality improvement (QI) initiatives, little work has been done to establish measures that organizations can use to monitor their improvement efforts.

**Objective:**

We sought to identify and evaluate existing OHL-related QI measures. To complement prior efforts to develop measures based on patient-reported data, we sought to identify measures computed from clinical, administrative, QI, or staff-reported data. Our goal was to develop a set of measures that experts agree are valuable for informing OHL-related QI activities.

**Methods:**

We used four methods to identify relevant measures computed from clinical, administrative, QI, or staff-reported data. We convened a Technical Expert Panel, published a request for measures, conducted a literature review, and interviewed 20 organizations working to improve OHL. From the comprehensive list of measures identified, we selected a set of high-priority measures for review by a second expert panel. Using a modified Delphi review process, panelists rated measures on four evaluation criteria, participated in a teleconference to discuss areas of disagreement among panelists, and rerated all measures.

**Key Results:**

Across all methods, we identified 233 measures. Seventy measures underwent Delphi Panel review. For 22 measures, there was consensus among panelists that the measures were useful, meaningful, feasible, and had face validity. Five additional measures received strong ratings for usefulness, meaningfulness, and face validity, but failed to show consensus among panelists regarding feasibility.

**Conclusions:**

We identified OHL-related QI measures that have the support of experts in the field. Although additional measure development and testing is recommended, the Consensus OHL QI Measures are appropriate for immediate use. **[*HLRP: Health Literacy Research and Practice*. 2019;3(2):e127–e146.]**

**Plain Language Summary:**

The health care system is complex. Health care organizations can make things easier for patients by making changes to improve communication and to help patients find their way around, become engaged in the health care process, and manage their health. We identify 22 measures that organizations can use to monitor their efforts to improve communication with and support for patients.

The United States health care system is complex and demanding. Patients and the families who help them must master a range of skills to manage their health successfully (DeWalt & McNeill, 2013). At a minimum, they must make appointments, navigate to and through health care facilities, comprehend written materials, articulate symptoms and answer questions, and understand and follow health care instructions. Successful completion of these tasks requires health literacy, defined as the “capacity to obtain, process, and understand basic health information and services needed to make appropriate health decisions” (Ratzan & Parker, 2000, p. vi). More than one-third of U.S. adults have limited health literacy skills (Kutner, Greenberg, Jin, & Paulsen, 2006). Such limitations are associated with poor health-related knowledge, self-care behavior, and outcomes (Berkman et al., 2004; Berkman, Sheridan, Donahue, Halpern, & Crotty, 2011; DeWalt & Hink, 2009).

Health care organizations can reduce the demands they place on patients and families. Organizational health literacy (OHL) is the degree to which an organization implements policies, practices, and systems that “make it easier for people to navigate, understand, and use information and services to take care of their health” (Brach et al., 2012, p. 1). In a recent review of theoretical frameworks and quality improvement (QI) resources, six factors were highlighted as critical components of OHL, including the importance of (1) enhancing communication with patients and families; (2) improving access to and navigation of health care facilities and systems; (3) encouraging patient engagement in the health care process; (4) establishing a workforce with OHL-related knowledge and skills; (5) creating an organizational culture and infrastructure supportive of OHL (e.g., commitment of leadership, development of appropriate policies); and (6) meeting patient needs, such as provision of interpreter services and self-management support (Farmanova, Bonneville, & Bouchard, 2018). The conceptual framework that guided this project incorporates these six factors, which are widely agreed to comprise OHL (Farmanova et al., 2018). Refined through consultation with the project's Technical Expert Panel (TEP), the framework organizes these concepts into four conceptual domains, each representing an area in which organizations can intervene to reduce demands on and improve support for patients and families (**Figure 1**). The Organizational Structure, Policy, & Leadership domain highlights the role of organization leaders in creating a culture committed to addressing health literacy. For instance, leaders may provide staffing for health literacy efforts, ensure providers receive training in OHL, show personal commitment to the organization's OHL initiatives, and support development of policies to improve communication, navigation, engagement, and self-management. The Communication domain consists of strategies organizations can use to enhance spoken, written, and cross-cultural communication, with the goal of improving comprehension of health information. The Ease of Navigation domain addresses strategies to simplify navigation of health care facilities (e.g., signage) and the health care system (e.g., simplifying referrals), making it easier for patients to access and use the care they need. Finally, the Patient Engagement & Self-Management Support domain encompasses strategies to enhance patient engagement in the health care process and system (e.g., establishing self-care goals, involving patients in organizational decision-making) and self-management capabilities (e.g., addressing nonmedical needs that can thwart optimal self-care, such as transportation barriers). Organizations implementing effective strategies in these domains can reduce demands and offer patients and families the additional support they may need to manage their health successfully.

Although numerous resources have been developed to help health care organizations improve OHL (Farmanova et al., 2018; Kripalani et al., 2014), only limited work has been done to establish measures that organizations can use to identify areas for improvement in OHL and to monitor the implementation and impact of OHL-related QI initiatives. Absent such measures, an organization may be unable to identify the features of its environment most in need of improvement or to determine whether OHL-related initiatives have been implemented effectively and have had the outcomes intended.

The objective of this project was to identify and to evaluate existing OHL-related QI measures, with the goal of establishing a set of measures supported by expert consensus. Consistent with the growing recognition that patient-reported outcome measures play an important role in performance evaluation (Basch, Torda, & Adams, 2013), earlier measure-development efforts focused on specification of OHL-related QI measures computed from patient survey data (Weidmer, Brach, & Hays, 2012; Weidmer, Brach, Slaughter, & Hays, 2012). These measures, which are part of the Consumer Assessment of Healthcare Providers and Systems (CAHPS), provide excellent insight into the adequacy of provider communication, for which the patient perspective is paramount.

To complement these measures, we sought to identify OHL-related QI measures computed from clinical or administrative data (e.g., electronic health record), QI data (i.e., data collected for the purpose of monitoring a QI effort), or staff-reported data (e.g., staff survey). Measures based on these data sources allow us to evaluate components of OHL that are less visible to patients (e.g., organizational policies regarding readability of written materials, OHL-related training requirements for staff). Likewise, these data sources enable development of process measures assessing the degree to which implementation of QI initiatives has been successful (e.g., percentage of providers trained to use the Teach-Back method for confirming patient understanding). In combination, measures that highlight the patient perspective and measures drawing on other data sources will allow for a more comprehensive assessment of OHL improvement.

## Methods

Project activities focused on (1) identifying existing OHL-related QI measures, (2) obtaining expert evaluation of a subset of these measures, and (3) establishing a set of Consensus OHL QI Measures that organizations can use to inform OHL-improvement efforts. The research protocol was approved by the Institutional Review Board of the University of Colorado Anschutz Medical Campus.

### Identification of Measures

We used four strategies to identify existing OHL-related QI measures. We (1) convened a TEP, (2) published a request for measures, (3) conducted a literature review, and (4) completed interviews with health care organizations engaged in OHL-related QI efforts.

*Technical Expert Panel*. In November 2015, we convened a TEP to obtain expert opinion on OHL and OHL-related measurement. Nine people with well-regarded experience implementing OHL-related QI initiatives served on the TEP (**Figure A**). Panelists provided input on the conceptual framework and identified existing OHL-related QI measures. To aid in later efforts to recruit organizations for interview participation, TEP members also identified organizations engaged in OHL-related QI efforts.

*Request for measures*. In February 2016, we published a request for information (RFI) in the Federal Register requesting nominations for OHL-related QI measures. We disseminated the RFI through national health literacy listservs as well as 28 state and regional health literacy programs. Some responses highlighted the OHL efforts of specific organizations, which were later considered for interview participation.

*Literature review*. We reviewed the peer-reviewed and grey literatures (i.e., sources not published through traditional academic or commercial publishers). In 2014, the Institute of Medicine (IOM; now The National Academy of Medicine) commissioned a literature review summarizing tools used to collect data or guide initiatives related to OHL (Kripalani et al., 2014). From this review, we isolated sources identifying OHL-related QI measures. With the assistance of a reference librarian, we updated the IOM review, refining its MEDLINE search strategy to capture additional concepts related to QI, OHL, and measurement (e.g., “quality improvement”). The search was performed using Ovid in March 2016.

In April 2016, we worked with a reference librarian to review the grey literature. Using key words consistent with our MEDLINE search (e.g., “health literacy,” “quality measures”), we explored online resources, such as conference proceedings and government reports. Websites targeted included those of Agency for Healthcare Research and Quality, Centers for Medicare & Medicaid Services, National Academy of Medicine, and National Quality Forum.

We screened titles and abstracts to identify resources describing OHL-related QI measures based on clinical, administrative, QI, or staff-reported data. The full text of relevant resources was obtained, and measures documented. In some cases, the literature highlighted organizations engaged in OHL-related QI efforts. These organizations were considered for interview participation.

*Organization interviews*. We conducted interviews with representatives of health care organizations working to improve OHL.

*Identification and prioritization of organizations*. As noted, the TEP, RFI, and literature review activities resulted in identification of relevant organizations. We also solicited organization nominations through health literacy listservs, state and regional health literacy programs, relevant medical boards, and interview participants. In addition, we identified organizations that participated successfully in an earlier OHL-related demonstration (Mabachi et al., 2016).

Eighty-two organizations were identified. To ensure detection of a broad range of measures, we prioritized organizations that were (1) actively engaged in implementing and measuring OHL-related QI efforts and (2) targeting multiple domains of OHL or a component of OHL not well addressed by other organizations. We sought to include a range of organization types, including primary care practices, clinics, hospitals, and health systems. We invited 21 organizations to participate in interviews.

*Data collection*. Twenty organizations agreed to participate. We conducted semi-structured interviews with knowledgeable representatives at each organization. Interviews followed a protocol designed to elicit detailed information about organizations' OHL-related measurement activities. So that interview participants would be comfortable sharing information about their experience conducting and evaluating OHL-related QI work, we assured interviewees that we would not publicly attribute their responses to them or their organizations in publications or presentations. During the interview, we requested any written documentation about the measures discussed. Using interview transcripts and written documentation, relevant QI measures were identified.

*Measure documentation*. For each measure identified that was computed from clinical, administrative, QI, or staff-reported data, we documented specific information. We recorded the measure title, description, and source; domain(s) targeted; computation specifications (e.g., data source, numerator, denominator); organizational settings in which the measure had been used; and psychometric testing results (when available).

### Evaluation of Measures

*Selection of measures for expert review*. We combined all measures identified into a comprehensive list of OHL-related QI measures. This list was culled to establish the “Candidate Measure Set,” which underwent expert review. In selecting Candidate Measures, we prioritized measures that (1) had potential to inform and aid in monitoring QI activities, (2) focused on recommended strategies for improving OHL (e.g., Teach-Back method) (Brega et al., 2015; Sheridan et al., 2012; Sudore & Schillinger, 2009; Weiss, 2007), and (3) were associated with commonly used health literacy resources (e.g., Health Literacy Environment of Hospitals and Health Systems; Rudd & Anderson, 2006). When duplicative measures were available, we selected the measure believed to be the strongest methodologically (e.g., prior psychometric testing, detailed computation specifications). We excluded measures that were proprietary or organization-specific, had weak or unclear specifications, targeted rare clinical scenarios, or were not clear indicators of OHL.

*Delphi Panel Review*. To obtain expert review of the Candidate Measures, we convened a Delphi Panel consisting of 10 people with complementary expertise in: (1) OHL, (2) quality measure development and evaluation, (3) implementation of OHL-related QI initiatives, and (4) patient-centered care (**Figure A**). To ensure that the patient perspective would be captured, the panel included a patient representative with quality measurement experience as well as four professionals with expertise in patient education, engagement, and/or measurement of patient- and family-centered outcomes. We used the RAND/UCLA Appropriateness Method (Fitch et al., 2001), a modified Delphi process, to obtain input on the Candidate Measures. **Table 1** provides information about the Delphi Panel review.

In the first step of the Delphi process, panelists independently reviewed and rated each Candidate Measure and provided written comments. Measures were rated on four evaluation criteria: usefulness, meaningfulness, face validity, and feasibility (see **Table 1** for definitions). Panelists used a five-point scale to rate the extent to which they agreed that the measures met each criterion (1 = *strongly disagree*, 2 = *somewhat disagree*, 3 = *neither agree nor disagree*, 4 = *somewhat agree*, and 5 = *strongly agree*).

After the initial review, we analyzed ratings and summarized written comments. For each measure, we computed a frequency distribution and median score for each criterion. We also assessed the degree of consensus in panelists' ratings. We classified ratings as showing consensus among panelists, a lack of consensus among panelists, or an inconclusive degree of consensus. The method for computing these classifications was based on the RAND/UCLA Appropriateness Method (Fitch et al., 2001), as refined to accommodate the size of the Delphi Panel and the 5-point rating scale (**Table 1**).

In May 2017, the TEP met via teleconference. Prior to the meeting, panelists received an aggregated summary of ratings, a confidential reminder of their own ratings, and a synthesis of written comments. Discussion at the meeting focused on measures for which ratings did not show consensus among panelists and measures that received strong ratings (median rating ≥4) on all criteria except feasibility. Our objective was to ensure panelists shared a consistent understanding of the measures and evaluation criteria. After the meeting, the eight panelists who had attended the teleconference independently rerated each measure. Again, we computed frequency distributions and median scores and classified the degree of consensus among panelists.

### Identifying Consensus OHL QI Measures

To be identified as a Consensus OHL QI Measure, a measure was required to meet two standards: (1) it had to have a median rating ≥4 for the usefulness, meaningfulness, face validity, and feasibility criteria and (2) ratings for each criterion had to show consensus among panelists.

## Results

### Measures Identified

Across all methods, we identified 233 measures. Most measures (56%) fell within the Communication domain, with 19% targeting the Ease of Navigation domain, 13% addressing the Patient Engagement & Self-Management Support domain, and 4% focusing on the Organizational Structure, Policy, & Leadership domain. Several measures (3%) were relevant to multiple domains and 5% focused on utilization metrics (mainly readmission) for which the domain of relevance would depend on the OHL strategy implemented.

### Consensus OHL QI Measures

Seventy measures were included in the Candidate Measure Set, which was reviewed by the Delphi Panel. Across these measures, 22 (31%) received strong ratings for usefulness, meaningfulness, face validity, and feasibility and showed consensus among panelists. These measures, classified as Consensus OHL QI Measures, are described in **Table 2**.

The Consensus OHL QI Measures cut across all OHL domains and a variety of measurement themes (**Table 3**). Eighteen percent of measures focus on the Organizational Structure, Policy, and Leadership domain, addressing themes such as leadership support for health literacy initiatives and implementation of structures to enhance patient engagement (e.g., dedicated staff). More than one-quarter of measures (27%) address the Communication domain. These measures focus on improving communication with patients having limited English proficiency, use of the Teach-Back method to improve patient comprehension of health information, and conduct of medication reviews to ensure accuracy and understanding of the medication regimen. Nine percent of measures target the Ease of Navigation domain, addressing strategies to simplify referrals and appointment scheduling. Nearly one-third of measures (32%) address the Patient Engagement & Self-Management Support domain. These measures target access to patient education, addressing patients' nonmedical needs, development of self-management goals, and provision of self-management support in the context of inpatient care. The remaining 14% of measures capture organizational performance across multiple domains.

Although all Consensus OHL QI Measures received support from the Delphi Panel, they vary in the degree to which they have previously undergone psychometric testing. As shown in **Table 2**, five measures have shown evidence of construct or face validity and/or reliability in previous investigations. Three of these measures received endorsement by the National Quality Forum, a nonprofit organization working to develop a national strategy for health care quality measurement. To our knowledge, the remaining measures have not undergone formal testing.

### Measures of Unclear Feasibility

Five Candidate Measures scored well (with consensus among panelists) on the usefulness, meaningfulness, and face validity criteria but failed to achieve consensus on feasibility (**Table 4**). In written comments and discussion during the teleconference, some panelists expressed concern that collection of the data needed to compute these measures was resource intensive. For instance, some panelists were concerned about the burden associated with staff surveys, which are required to compute measures based on the Communication Climate Assessment Toolkit. Likewise, some panelists questioned the feasibility of a measure assessing the impact of health literacy training on provider skills due to concern about the time required to train assessors and conduct staff observations.

## Discussion

Although numerous toolkits and resources have been developed to guide the efforts of health care organizations seeking to improve OHL (Farmanova et al., 2018; Kripalani et al., 2014), related measure-development work has been limited. Through this effort, we established a set of 22 measures that experts agreed have face validity and are useful, meaningful, and feasible for monitoring and informing OHL-related QI initiatives. Five additional measures were well rated regarding usefulness, meaningfulness, and face validity, but received inconsistent ratings for feasibility, as a result of concerns about staff time required to collect the data underlying these measures. It is likely that larger health care organizations and those that have an existing infrastructure to support routine data collection may find these measures more manageable. For other organizations, it may be possible to identify strategies that would make adoption of these measures feasible (e.g., involving volunteers in data collection, providing time during staff meetings to complete surveys).

Development of the Consensus OHL QI Measures represents an important step in the national agenda to improve OHL (Adams & Corrigan, 2003; Carmona, 2006; Kindig, Panzer, & Nielsen-Bohlman, 2004; Koh et al., 2012; Office of Disease Prevention and Health Promotion, 2010; U.S. Department of Health and Human Services, 2000). As a complement to previously developed CAHPS measures assessing patient perceptions of provider communication (Weidmer, Brach, & Hays, 2012; Weidmer, Brach, Slaughter, et al., 2012), the Consensus OHL QI Measures offer organizations measures that target a wider array of OHL concepts. Across the Consensus OHL QI Measures, each of the four domains of OHL is addressed, as are 12 important measurement themes. As an added benefit, because the measures are derived from clinical, administrative, QI, or staff-reported data, they impose no burden on patients.

Measurement burden is a concern in the U.S. health care system. Health care organizations routinely collect data related to payment, accreditation, and clinical performance (Dunlap et al., 2016; Institute of Medicine, 2015). The Consensus OHL QI Measures are meant to support an organization's internal efforts to improve OHL. That said, organizations may find that implementing OHL-related QI initiatives can further their progress toward regulatory requirements or other organizational aims. For instance, health care practices seeking certification as Patient-Centered Medical Homes will find concepts central to OHL (e.g., effective communication, support for patient engagement and self-management) to be critical to patient-centered care (Agency for Healthcare Research and Quality, n.d.). Likewise, organizations receiving value-based payments that reward positive outcomes may benefit from efforts to make health information more understandable, to simplify navigation of the health care system, and to support patient engagement and self-care (Brach, 2017). OHL initiatives can complement these other organizational priorities, with the Consensus OHL QI Measures serving to support the process.

Although the Consensus OHL QI Measures provide an important resource, they have limitations. Despite the breadth of domains and themes addressed, some important concepts are not captured (e.g., written communication, navigating an organization's physical environment). Further, although it is possible that some measures have undergone testing of which we are unaware (e.g., unpublished testing conducted by the health care organizations that developed the measures), we were able to locate evidence of prior psychometric testing for only five of the Consensus OHL QI Measures. Unlike accountability measures, however, QI measures often do not undergo rigorous testing and the Consensus OHL QI Measures have the benefit of having the support of experts in the field. Finally, some Consensus OHL QI Measures identify whether a process has occurred but not whether it followed best practices or had the desired effect. For instance, Consensus Measure (CM)-10 (**Table 2**) captures the percentage of older adults for whom a medication review was completed. It does not assess whether the review was conducted in accordance with recommended practices (e.g., use of Teach Back) nor whether it resulted in improved patient comprehension of the medication regimen.

Future measure-development efforts should aim to address these limitations, generating measures to fill the gaps in the current set of measures and conducting additional psychometric testing. In the next stage of OHL measure development, we suggest systematic identification or generation of “companion measures” that, together, can capture both the implementation and impact of OHL efforts. The Consensus OHL QI Measures include some examples of companion measures. For instance, measure CM-8 captures the percentage of staff members trained to use Teach Back and measure CM-9 captures the percentage of patients who can teach back their discharge instructions correctly. Together, these measures evaluate how effectively a QI initiative was implemented and whether it had the desired effect. Valuable companion measures could be developed for many of the Consensus OHL QI Measures, enhancing the ability of organizations to evaluate both the implementation and outcomes of their QI initiatives.

## Conclusion

In conclusion, this systematic effort to identify and evaluate existing OHL-related QI measures represents an important step forward in the effort to improve OHL. The Consensus OHL QI Measures can provide a valuable resource for health care organizations seeking to make it easy for patients and their families to navigate, understand, and use information and services to take care of their health. We recommend that future measure-development efforts generate additional QI measures targeting themes and constructs that are not adequately addressed by the Consensus OHL QI Measures, that measure developers systematically aim to capture both the process and outcomes of OHL QI efforts, and that additional psychometric testing be conducted. Until a more comprehensive set of measures becomes available, we encourage organizations to use the Consensus OHL QI Measures to inform their OHL-improvement efforts.

## Figures and Tables

**Figure 1.**
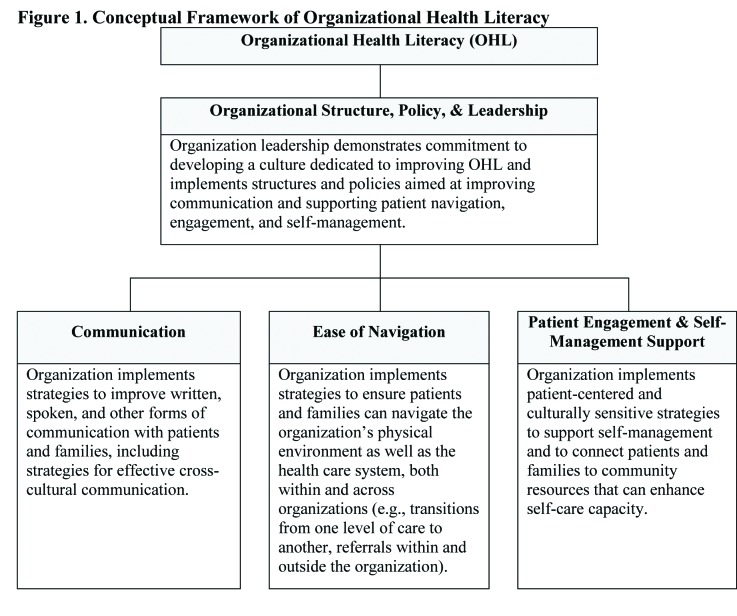
Conceptual framework of organizational health literacy.

**Table 1:** Delphi Panel Review

**Key steps in the review process** *Step 1*. Panelists independently reviewed and rated each measure on four criteria and provided written comments *Step 2*. We analyzed ratings, synthesized comments, and provided summary findings to panelists *Step 3*. Panel met by teleconference to discuss measures for which ratings did not show consensus among panelists and measures with strong ratings for all criteria except feasibility *Step 4*. Panelists independently rerated each measure on four criteria and provided written comments **Evaluation criteria used in Delphi Panel Review** *Usefulness*: The measure can be used to monitor and inform quality improvement efforts aimed at improving organizational health literacy *Meaningfulness*: The measure assesses a component of organizational health literacy that is meaningful to key stakeholders (e.g., patients, clinicians, administrators) *Face validity*: The measure appears to capture the construct it is designed to assess *Feasibility*: The measure can be computed with accuracy and implemented in a timely manner, without undue burden **Classifying the degree of consensus among panelists** *Consensus*: ≤2 ratings deviated from the median score by ≥1.5 points *Lack of consensus*: ≥3 ratings occurred in each tail of the rating scale (i.e., ≥3 ratings of 1 or 2 and ≥3 ratings of 4 or 5) *Inconclusive*: Ratings did not meet the criteria for consensus or lack of consensus

**Table 2:** Consensus Organizational Health Literacy Quality Improvement Measures

**Consensus Measure Number, Title, and Description**	**Measure Source,^a^ Data Source, Measure Computation Specifications, and Health Care Setting^b^**	**Psychometric Testing and National Endorsement**
OHL Domain: Organizational Structure, Policy, & Leadership		
Measurement theme: Leadership support for organizational health literacy activities		
Number: CM-1 Title: Leadership Support of Health Literacy Efforts Description: Percentage of leaders who attended health literacy awareness activity	Measure source: Health care organization Data source: Process data collected by implementation staff Numerator: Number of members of the organization's senior leadership (e.g., medical director, chief executive officer, nursing manager) who attend health literacy awareness activity Denominator: Number of members of the organization's senior leadership Setting: Measure is relevant across settings	None identified
Measurement theme: Staffing and structures to enhance patient and family engagement		
Number: CM-2 Title: PFE Hospital Evaluation Metric 3—PFE Leader or Functional Area^c^ Description: Hospital has a person or functional area, who may also operate within other roles in the hospital, that is dedicated and proactively responsible for Patient & Family Engagement and systematically evaluates PFE activities (i.e., open chart policy, PFE trainings, establishment and dissemination of PFE goals)	Measure source: American Institutes for Research (2016) Data source: Organization leadership (e.g., chief quality officer, vice president for patient experience) can report whether policy exists Computation: Measure assesses whether the organization has a person or unit that is responsible for initiating and evaluating patient and family-engagement activities Setting: Designed for hospitals, but relevant across settings	The Centers for Medicare & Medicaid Services uses this measure as 1 of 5 metrics aimed at supporting efforts to improve PFE (American Institutes for Research, 2016). We were unable to identify prior psychometric testing
Measurement theme: Structured methods for encouraging PFE		
Number: CM-3 Title: PFE Hospital Evaluation Metric 4-Patient and Family Advisory Council or Representative on Quality Improvement Team^c^ Description: Hospital has an active Patient and Family Engagement Committee (PFEC) or at least one former patient that serves on a patient safety or quality improvement committee or team	Measure source: American Institutes for Research (2016) Data source: Organization leadership (e.g., chief quality officer, vice president for patient experience) can report whether policy exists Computation: Measure assesses whether the organization (1) has a PFE Committee or (2) involves at least one former patient on a patient safety or quality improvement committee Setting: Designed for hospitals, but relevant across settings	The Centers for Medicare & Medicaid Services uses this measure as 1 of 5 metrics aimed at supporting efforts to improve PFE (American Institutes for Research, 2016). We were unable to identify prior psychometric testing
Number: CM-4 Title: PFE Hospital Evaluation Metric 5 – Patient(s) and Family on Hospital Governing and/or Leadership Board^c^ Description: Hospital has at least one or more patient(s) who serve on a Governing and/or Leadership Board and serves as a patient representative	Measure source: American Institutes for Research (2016) Data source: Organization leadership (e.g., chief quality officer, vice president for patient experience) can report whether policy exists Computation: Measure assesses whether the organization has at least one patient serving as a representative on the organization's governing or leadership board Setting: Designed for hospitals, but relevant across settings	The Centers for Medicare & Medicaid Services uses this measure as 1 of 5 metrics aimed at supporting efforts to improve PFE (American Institutes for Research, 2016). We were unable to identify prior psychometric testing
OHL Domain: Communication		
Measurement theme: Serving patients with limited English proficiency		
Number: CM-5 Title: Screening for Preferred Spoken Language for Health Care Description: Percentage of hospital admissions, visits to the emergency department, and outpatient visits for which preferred spoken language for health care is identified and recorded	Measure source: National Quality Forum (2012f) Data source: Claims data, electronic health record/medical chart Numerator: Number of hospital admissions, visits to the emergency department, and outpatient visits during which patient's preferred spoken language for health care is identified and recorded Denominator: Number of hospital admissions, visits to the emergency department, and outpatient visits Setting: Hospitals and other inpatient facilities, and urgent care	This measure has shown evidence of face and construct validity (National Quality Forum, 2012b) and has been incorporated into the Agency for Healthcare Research and Quality's National Measures Clearinghouse. Although the measure received initial endorsement by the National Quality Forum (Measure 1824 L1A), endorsement was removed in April 2017 (National Quality Forum, n.d.). According to J. Tilly of the National Quality Forum (personal communication, June 28, 2018), endorsement was removed because the Measure Steward was longer interested in maintaining the measure, not due to concerns over the measure's scientific acceptability
Number: CM-6 Title: Patients Receiving Language Services Supported by Qualified Language Services Providers Description: Percentage of patients who state a preference to receive spoken health care in a language other than English who have documentation in their electronic health record that they received initial assessment and discharge instructions supported by trained and assessed interpreters or bilingual providers, workers, or employees assessed for language proficiency	Measure source: National Quality Forum (2012f) Data source: Electronic health record/medical chart Numerator: Number of patients with limited English proficiency for whom the electronic health record documents that the patient received initial assessment and discharge instructions supported by trained and assessed interpreters or from bilingual providers, workers, or employees assessed for language proficiency Denominator: Number of patients who stated a preference to receive spoken health care in a language other than English Exclusions: Patients who state a preference to receive spoken health care in English, leave without being seen, or leave against medical advice prior to initial assessment Setting: Hospitals and other inpatient facilities, and urgent care	This measure has shown evidence of face and construct validity (National Quality Forum, 2012b) and has been incorporated into the Agency for Healthcare Research and Quality's National Measures Clearinghouse. Although the measure received initial endorsement by the National Quality Forum (Measure 1821 L2), endorsement was removed in April 2017 (National Quality Forum, n.d.). According to J. Tilly of the National Quality Forum (personal communication, June 28, 2018), endorsement was removed because the Measure Steward was no longer interested in maintaining the measure, not due to concerns over the measure's scientific acceptability
Number: CM-7 Title: Patients Receiving Language Services During Consent Discussions Description: Percentage of informed consent discussions for patients with limited English proficiency that have documentedinvolvement of an interpreter	Measure source: Health care organization Data source: Electronic health record/medical chart Numerator: Number of patients with limited English proficiency for whom the consent discussion involved an interpreter Denominator: Number of patients with limited English proficiency who had an informed consent discussion Setting: Measure is relevant across settings	None identified
Measurement theme: Using the Teach-Back method to ensure patient comprehension		
Number: CM-8 Title: Staff Trained to Use Teach Back Description: Percentage of staff who report being formally trained to use the Teach-Back method	Measure source: Health care organization Data source: Staff survey item: “Have you been formally trained to use the Teach-Back technique?” Response Options: *yes*, *partially*, *no* Numerator: Number of staff members who answer “*yes”* when asked if they have received formal training in using the Teach-Back method Denominator: Number of staff who completed the staff survey Setting: Measure is relevant across settings	None identified
Number: CM-9 Title: Patients Correctly Teaching Back Discharge Instructions Description: Percentage of discharged patients who correctly taught back discharge instructions	Measure source: Health care organization Data source: Electronic health record/medical chart Numerator: Number of patients for whom the electronic health record documents that Teach Back was conducted and that the patient was able to correctly teach back discharge instructions Denominator: Number of patients discharged Setting: Hospitals and other inpatient facilities	None identified
Measurement theme: Medication review to improve accuracy and patient understanding		
Number: CM-10 Title: Care for Older Adults – Medication Review Description: Percentage of adults 66 years and older who had a medication review	Measure source: National Quality Forum (2010) Data source: Electronic health record/medical chart Numerator: Number of patients with at least one medication review conducted by a prescribing practitioner or clinical pharmacist during the measurement year and the presence of a medication list in the medical record Denominator: All patients age 66 years and older as of December 31 of the measurement year Setting: Hospitals and other inpatient facilities, ambulatory care, post-acute care	This measure has shown strong evidence of reliability (National Quality Forum, 2012a) and has been endorsed by the National Quality Forum (Measure 0553) since August 2009 (National Quality Forum, n.d.)
OHL Domain: Ease of Navigation		
Measurement theme: Simplifying the process of scheduling appointments		
Number: CM-11 Title: Follow-up Appointment Scheduling Description: Percentage of patients who get follow-up appointments made upon discharge	Measure source: Health care organization Data source: Electronic health record/medical chart Numerator: Number of patients for whom a follow-up appointment is made prior to discharge Denominators: Number of patients discharged Setting: Hospitals and other inpatient facilities	None identified
Measurement theme: Ensuring referral completion		
Number: CM-12 Title: Referral Report Received Description: Number of patients with a referral for whom the referring provider received a follow-up report from the provider to whom the patient was referred	Measure source: Health care organization Data source: Electronic health record/medical chart Computation: Number of patients with a referral for whom the referring provider received a follow-up report describing the results of the referral visit Setting: Ambulatory care, health systems	None identified
OHL Domain: Patient Engagement & Self-Management Support		
Measurement theme: Improving access to patient education		
Number: CM-13 Title: Inpatient Education Received Description: Percentage of inpatients given patient education on bedside tablet who complete the education module	Measure source: Health care organization Data source: Electronic health record/medical chart or process data collected by implementation staff Numerator: Number of inpatients who complete patient education using bedside tablet Denominator: Number of inpatients offered patient education using bedside tablet Setting: Hospitals and other inpatient facilities	None identified
Measurement theme: Addressing patients' nonmedical needs		
Number: CM-14 Title: Screening for Nonmedical Needs Description: Percentage of patients screened for nonmedical needs	Measure source: Health care organization Data source: Electronic health record/medical chart Numerator: Number of patients screened for nonmedical needs (e.g., housing, transportation, food assistance) Denominator: Number of patients Setting: Measure is relevant across settings	None identified
Number: CM-15 Title: Referral for Nonmedical Needs Description: Percentage of patients who screened positive for needing nonmedical support who were referred for services	Measure source: Health care organization Data source: Electronic health record/medical chart Numerator: Number of patients referred for nonmedical services (e.g., housing, transportation, food assistance) Denominator: Number of patients who “screened positive” for having nonmedical needs Setting: Measure is relevant across settings	None identified
Measurement theme: Setting self-management goals		
Number: CM-16 Title: Self-Management Goals Description: Percentage of patients with diabetes who have set a self-management goal	Measure source: Health care organization Data source: Electronic health record/medical chart Numerator: Number of patients with diabetes who have a self-management goal documented in the electronic health record or medical chart Denominator: Number of patients with diabetes Setting: Ambulatory care	None identified
Measurement theme: Self-management support before, during, and after an inpatient stay		
Number: CM-17 Title: PFE Hospital Evaluation Metric 1—Planning Checklist for Scheduled Admissions Description: Prior to admission, hospital staff provide and discuss a discharge-planning checklist with every patient who has a scheduled admission, allowing for questions or comments from the patient or family (e.g., a planning checklist that is similar to the Centers for Medicare & Medicaid Service's Discharge Planning Checklist)	Measure source: American Institutes for Research (2016) Data source: Organization leadership (e.g., chief quality officer, vice president for patient experience, director of nursing) can report whether policy exists Computation: Measure assesses whether the organization has a policy to review a discharge-planning checklist with all patients prior to admission Setting: Designed for hospitals, but relevant across inpatient settings	The Centers for Medicare & Medicaid Services uses this measure as 1 of 5 metrics aimed at supporting efforts to improve PFE (American Institutes for Research, 2016). We were unable to identify prior psychometric testing
Number: CM-18 Title: PFE Hospital Evaluation Metric 2—Shift Change Huddles/Bedside Reporting Description: Hospital conducts shift change huddles for staff and does bedside reporting with patients and family members in all feasible cases	Measure source: American Institutes for Research (2016) Data source: Organization leadership (e.g., chief quality officer, vice president for patient experience, director of nursing) can report whether policy exists Computation: Measure assesses whether the organization has a policy to conduct shift change huddles for staff and bedside reporting with patients and families Setting: Designed for hospitals, but relevant across inpatient settings	The Centers for Medicare & Medicaid Services uses this measure as 1 of 5 metrics aimed at supporting efforts to improve PFE (American Institutes for Research, 2016). We were unable to identify prior psychometric testing
Number: CM-19 Title: Postdischarge Phone Call Description: Percentage of discharged patients for whom postdischarge phone call was completed	Measure source: Auerbach et al. (2014) Data source: Electronic health record/medical chart Numerator: Number of discharged patients who received a postdischarge phone call Denominator: Number of discharged patients who were supposed to receive a postdischarge phone call Setting: Hospitals and other inpatient facilities, and urgent care	None identified
Measures that cut across domains		
Number: CM-20 Title: Health Literate Health Care Organization-10 (HLHO-10) Score Description: Computed score based on hospital administrator's responses to 10 questions designed to assess the 10 attributes of a health literate health care organization	Measure source: Kowalski et al. (2015) Data source: Survey of Hospital Administrator (Kowalski et al., 2015) Computation: Administrator responds to 10 questions using a 7-point scale ranging from *not at all*(1) to *to a very large extent*(7). The overall score is the mean score across the 10 items Setting: Hospitals	Survey tested with 51 German hospitals and found to have strong internal consistency reliability (α = 0.89) and to significantly predict breast cancer patients' perceptions of the adequacy of health information received (Kowalski et al., 2015)
Number: CM-21 Title: Health Literate Discharge Score Description: Computed score based on staff responses to 36 questions addressing language preferences/needs, communication regarding needed follow-up appointments, medication review, readability of written care plan, patient education, and follow-up after discharge	Measure source: Innis, Barnsley, Berta, & Daniel (2017) Data source: Staff Survey (Innis et al., 2017) Computation: Staff respond to 36 questions using a 5-point Likert scale. For each respondent, the mean score across items is computed. The overall score is the mean score across respondents (range, 36–180) Setting: Hospitals	Survey was tested with nursing managers and other staff from 79 hospitals in Canada. Four of the five factors on which the items loaded showed strong internal consistency reliability (α = 0.80–0.91), with one factor just missing the usual threshold for establishing adequate reliability (α = 0.68) (Innis et al., 2017)
Number: CM-22 Title: Overall Health Literacy Environment Rating Description: Sum of 5 domain scores based on Health Literacy Environment Review: navigation, print communication, oral exchange, technology, and policies and protocols	Measure source: Rudd & Anderson (2006) Data source: Staff assessment using Health Literacy Environment Review (Rudd & Anderson, 2006) Computation: Sum of print communication rating, technology rating, oral exchange rating, navigation rating, and policies and protocols rating Setting: Hospitals and other inpatient facilities, ambulatory care	None identified

Note. CM = consensus measure; OHL = organizational health literacy; PFE = person and family engagement.

a Measures identified through interviews with health care organizations working to improve their OHL are identified as having a Measure Source of “health care organization.” Because we assured participants in the organization interviews that their responses would remain confidential, we do not identify health care organizations by name.

b Setting refers to the health care settings for which a measure is believed to be relevant (e.g., hospitals).

c Although the PFE Hospital Evaluation Metrics were designed to assess engagement, we have categorized 3 of the 5 measures as addressing the Organizational Structure, Policy, & Leadership domain. For each of these measures, improved engagement is pursued through implementation of organizational structures and policies (i.e., staffing to support patient engagement efforts, patient involvement in committees).

**Table 3:** Domains and Themes Addressed by Consensus Organizational Health Literacy Quality Improvement Measures

**Organizational Health Literacy Domain and Measurement Theme**	**Number of Consensus Measures (%)^a^**

Organizational Structure, Policy, & Leadership	4 (18%)
Leadership support for organizational health literacy activities	1 (5%)
Staffing and structures to enhance patient and family engagement	1 (5%)
Structured methods for encouraging patient and family engagement	2 (9%)

Communication	6 (27%)
Serving patients with limited English proficiency	3 (14%)
Using the Teach-Back method to ensure patient comprehension	2 (9%)
Medication review to improve accuracy and patient understanding	1 (5%)

Ease of Navigation	2 (9%)
Simplifying the process of scheduling appointments	1 (5%)
Ensuring referral completion	1 (5%)

Patient Engagement & Self-Management Support	7 (32%)
Improving access to patient education	1 (5%)
Addressing patients' nonmedical needs	2 (9%)
Setting self-management goals	1 (4%)
Self-management support before, during, and after an inpatient stay	3 (14%)

Measures that cut across domains	3 (14%)

Note.

a Because of rounding error, percentages related to each measurement theme may not sum to the total percentage of measures within a given domain.

**Table 4:** Supplemental Measures with Unclear Feasibility

**Measure Title and Description**	**Measure Source,**^a^ **Data Source, Measure Computation Specifications, and Health Care Setting**^b^	**Psychometric Testing and National Endorsement**
OHL Domain: Communication		
Measurement theme: Health literacy-related training for staff		
Title: Impact of Health Literacy Training on Skill Development Description: Percentage of staff members attending health literacy training who are able to role play health literacy strategies (e.g., use of Teach Back)	Measure source: Health care organization Data source: Process data collected by implementation staff Numerator: Number of staff members who are able to adequately role play health literacy strategies (e.g., use of Teach Back) Denominator: Number of staff members attending health literacy training Setting: Measure is relevant across settings	None identified
Title: Communication Climate Assessment Toolkit Workforce Development Domain Description: Computed score based on staff responses to 21 questions assessing whether organization provides adequate training in spoken communication	Measure source: Wynia, Johnson, McCoy, Griffin, and Osborn (2010) Data source: Staff Survey (University of Colorado Center for Bioethics and Humanities, 2018). Must obtain responses from at least 50 clinical and nonclinical staff members Computation: Responses to each item are coded using a 0–1 scale, with 1 being the desirable response. For each respondent, the average score across survey items addressing this domain is calculated. The average of these scores across respondents is then calculated and multiplied by 100, resulting in a score between 0 and 100 Exclusions: Staff members who do not have direct contact with patients are excluded from questions that target patient contact Setting: Hospitals and clinics	A version of this measure has been endorsed by the National Quality Forum (Measure 1888) (National Quality Forum, 2012d). The endorsed measure includes both patient and staff survey data. Because we focused on measures derived from clinical, administrative, quality improvement, or staff-reported data, the measure presented here only includes staff survey data. Although the staff survey items have shown strong internal consistency reliability (α = 0.93) (Wynia et al., 2010), psychometric testing of a measure using only staff survey data is recommended
Measurement theme: Monitoring and improvement of communication		
Title: Communication Climate Assessment Toolkit Performance Evaluation Domain Description: Computed score based on staff responses to 7 questions about the degree to which the organization regularly monitors and seeks to improve the quality of communications with patients and among hospital/clinic staff	Measure source: Wynia et al. (2010) Data source: Staff Survey (University of Colorado Center for Bioethics and Humanities, 2018). Must obtain responses from at least 50 clinical and nonclinical staff members Computation: Responses to each item are coded using a 0–1 scale, with 1 being the desirable response. For each respondent, the average score across survey items addressing this domain is calculated. The average of these scores across respondents is then calculated and multiplied by 100, resulting in a score between 0 and 100 Exclusions: Staff members who do not have direct contact with patients are excluded from questions that target patient contact Setting: Hospitals and clinics	A version of this measure has been endorsed by the National Quality Forum (Measure 1901) (National Quality Forum, 2012e). The endorsed measure includes both patient and staff survey data. Because we focused on measures derived from clinical, administrative, quality improvement, or staff-reported data, the measure presented here only includes staff survey data. Although the staff survey items have shown strong internal consistency reliability (α = 0.84) (Wynia et al., 2010), psychometric testing of a measure using only staff survey data is recommended
Measurement theme: Serving patients with limited English proficiency		
Title: Communication Climate Assessment Toolkit Data Collection Domain Description: Computed score based on staff responses to 9 questions assessing whether organization collects information on patient demographics and interpretation needs	Measure Source: Wynia et al. (2010) Data Source: Staff Survey (University of Colorado Center for Bioethics and Humanities, 2018). Must obtain responses from at least 50 clinical and nonclinical staff members. Computation: Responses to each item are coded using a 0–1 scale, with 1 being the desirable response. For each respondent, the average score across survey items addressing this domain is calculated. The average of these scores across respondents is then calculated and multiplied by 100, resulting in a score between 0 and 100 Exclusions: Staff members who do not have direct contact with patients are excluded from questions that target patient contact Setting: Hospitals and clinics	A version of this measure has been endorsed by the National Quality Forum (Measure 1881) (National Quality Forum, 2012c). The endorsed measure includes both patient and staff survey data. Because we focused on measures derived from clinical, administrative, quality improvement, or staff-reported data, the measure presented here only includes staff survey data. Although the staff survey items have shown strong internal consistency reliability (α = 0.90) (Wynia et al., 2010), psychometric testing of a measure using only staff survey data is recommended
Title: Interpreter Use During Inpatient Stay Description: Number of encounters per inpatient stay for which a patient with a language preference other than English had the necessary/appropriate interpreter present	Measure Source: Health care organization Data Source: Electronic health record/medical chart Numerator: Number of encounters involving on-site, telephone, or video interpreters Denominator: Number of inpatient stays of patients with a language preference other than English Setting: Hospitals and other inpatient facilities	None identified

Note.

a Measures identified through interviews with health care organizations working to improve their organizational health literacy are identified as having a Measure Source of “health care organization.” Because we assured participants in the organization interviews that their responses would remain confidential, we do not identify health care organizations by name.

b Setting refers to the health care settings for which a measure is believed to be relevant (e.g., hospitals).

**Figure A.**
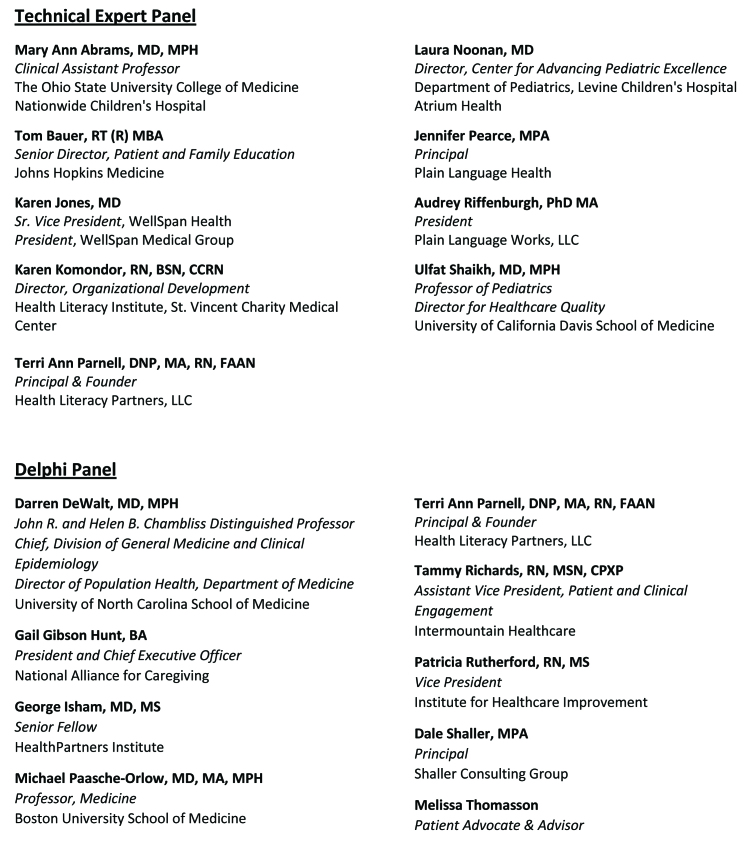
Technical Expert Panel and Delphi Panel members.
